# Regulatory T Cells in Colorectal Cancer: From Biology to Prognostic Relevance

**DOI:** 10.3390/cancers3021708

**Published:** 2011-03-29

**Authors:** Dimitrios Mougiakakos

**Affiliations:** Department of Oncology and Pathology, Immune and Gene Therapy Unit, Cancer Centre Karolinska, CCK R8:01, 17176 Stockholm, Sweden; E-Mail: dimitrios.mougiakakos@ki.se

**Keywords:** regulatory T cells, colorectal cancer, prognostic marker, immune escape

## Abstract

Regulatory T cells (Tregs) were initially described as “suppressive” lymphocytes in the 1980s. However, it took almost 20 years until the concept of Treg-mediated immune control in its present form was finally established. Tregs are obligatory for self-tolerance and defects within their population lead to severe autoimmune disorders. On the other hand Tregs may promote tolerance for tumor antigens and even hamper efforts to overcome it. Intratumoral and systemic accumulation of Tregs has been observed in various types of cancer and is often linked to worse disease course and outcome. Increase of circulating Tregs, as well as their presence in mesenteric lymph nodes and tumor tissue of patients with colorectal cancer *de facto* suggests a strong involvement of Tregs in the antitumor control. This review will focus on the Treg biology in view of colorectal cancer, means of Treg accumulation and the controversies regarding their prognostic significance. In addition, a concise overview will be given on how Tregs and their function can be targeted in cancer patients in order to bolster an inherent immune response and/or increase the efficacy of immunotherapeutic approaches.

## 1. Introduction

From the very beginnings of cancer research, it has been postulated that the immune system plays an important role in controlling tumor development and progression. The concept of immune cells efficiently recognizing and destroying neoplastic cells is known as tumor-immunosurveillance [1]. One key and at first glance contradictory finding has been that although activated immune cells represent an important component of the cancer microenvironment, they regularly fail to elicit an efficient disease control. The majority of these cells is specific for a number of tumor-associated antigens (TAAs), which have emerged as being normal self-constituents [2]. This repeatedly made observation implicates that tumor immunity partly resembles autoimmunity. As a consequence understanding self-tolerance became obligatory in order to unscramble the mechanisms underlying tumor-tolerance. Multiple mechanisms contribute to the establishment and maintenance of self-tolerance. The thymus, where self-reactive T cells are efficiently deleted during negative selection, is of central relevance [3]. Nevertheless, self-reactive T cells still can be found in the periphery where they normally fail to get activated due to their low avidity T cell receptor (TCR) or the lack of co-stimulation by self-antigen presenting cells. For a long time it was hypothesized that these passive mechanisms of self-protection are complemented by a dominant cellular control [4].

In the 1970s Gershon and Kondo reported [5,6] the existence of thymus-derived lymphocytes that suppressed an antigen-induced T cell activation. In great foresight they named this population “suppressor cells”, but several attempts to isolate and further characterize them failed due to the lack of a specific phenotype. Almost twenty years later Sakaguchi *et al.* assigned them their first phenotype as CD4^+^CD25^+^ T cells [7]. Depleting these cells in mice resulted in spontaneous autoimmunity, which was successfully resolved upon their re-infusion. These findings ultimately heralded the era of major research efforts on Tregs. Nowadays the most established phenotype for Tregs is defined by the expression of CD25 [8,9] and FOXP3 [10,11]. Unfortunately both are not exclusive markers as they can also be found in activated conventional T cells (Tconv), albeit at lower levels [8,12]. The surface molecule CD25 is the α-chain of a high-affinity IL-2 receptor whereas the forkhead/winged transcription factor (TF) FOXP3 represents the master regulator for Treg ontogeny and function [13,14].

Tregs comprise 5%–10% of the total peripheral CD4^+^ T cells [8] and it is generally believed that they hold a key role in maintaining self-tolerance by dominantly suppressing the activation as well as the function of (especially self-reactive) lymphocytes [15]. Defects of the Treg compartment impair immune homeostasis as characteristically seen in the patients with IPEX (Immunodysregulation, Polyendocrinopathy, Enteropathy, X-linked) syndrome but also systemic lupus erythematosus or multiple sclerosis [13,15-17]. Already at the time of their emergence, the vaguely characterized “suppressor cells” were regarded as a double-edged sword in the context of malignant diseases. Fujimoto and North demonstrated that suppressive lymphocytes were present in tumor bearing mice and efficiently suppressed the rejection of even highly immunogenic chemically induced fibrosarcomas [18-20].

The means of analyzing and isolating Tregs in cancer patients naturally followed the “evolutionary path” of their progressing phenotypic characterization [21]; soon after CD4^+^CD25^high^ lymphocytes were convincingly shown to grossly represent the suppressive population in healthy individuals [8] it could be demonstrated that such cells were infiltrating tumor tissue and circulated at increased proportions in patients with lung and ovarian cancer [22]. As soon as FOXP3 proved to be a more specific Treg marker in 2003 it was implemented into the current analysis panels as firstly successfully shown in patients with ovarian cancer by Tyler J. Curiel and colleagues [23].

This review will focus on the biology and the role of Tregs in colorectal cancer as one of the leading causes of morbidity and mortality [24]. Furthermore, we will discuss their impact on disease development and progression as well as the prospects of targeting them in therapeutic interventions.

## 2. Major Subsets, Immunological Features and Accumulation in Cancer

### 2.1. Major Subsets

In the past decade various phenotypes and functions have been allocated to distinct Treg subsets. These populations include both, CD4^+^ as well as CD8^+^ cells and even CD4^−^CD8^−^ double negative (DN) variants [25]. Based on their ontogeny, Tregs have been dichotomized into two major, from the genomic standpoint distinct, subsets [26]: the natural form (nTregs) arising from the thymus and subsequently populating the periphery and the induced cells (iTregs), which are generated by a conversion of Tconv. This peripheral transformation occurs under various conditions and most times requires a preceding MHC-peptide interaction. Due to the involution of the thymus with age Treg output declines over time but still remains detectable [27].

To date naturally occurring CD4^+^CD25^+^FOXP3^+^ Tregs (nTregs) have been the most extensively studied subset. Characteristically they constitutively express CD25, glucocorticoid induced TNFR family-related protein (GITR) and cytotoxic T lymphocyte antigen (CTLA-4), which are all under the control of FOXP3. Two recent studies demonstrated that the IL-7 receptor α-chain (CD127) could be useful for the discrimination of nTregs from activated Tconvs [28,29]. Since FOXP3 expression correlates inversely with CD127, nTregs consequently depict no or low CD127 levels on their surface. Thus combination of CD127 with CD25 expression (CD3^+^CD4^+^CD25^+^CD127^low/neg^) allows a more elaborate identification and even purification of viable Tregs.

The cytokine composition of the milieu, in which Tconvs receive their activating stimuli, plays a decisive role for iTreg generation. A repetitive, antigen-dependent stimulation of naïve or memory CD4^+^ Tconvs in presence of primarily IL-10 (but also vitamin D, dexamethasone, complement factor CD3b and CD4b) leads to the generation of mainly IL-10 producing FOXP3^+^ suppressive cells (T regulatory cells 1; Tr1) [30-32]. Similarly the presence of TGF-β results into the induction of cells that produce high amounts of TGF-β (T helper cells 3; Th3) and can express FOXP3 but not as stably as nTregs [26,33,34]. Once activated by a specific antigen, iTregs do not require additional re-stimulation and suppress in an antigen-independent fashion. This characteristic led to the term “bystander suppression”. The TCR repertoire of iTregs is congruent with T4convs and partly overlaps with nTregs [35]. Other (tumor derived) molecules that have been linked to iTregs include prostaglandin E2 (PGE_2_), indoleamine 2,3-dioxygenase (IDO) and Galectin-1 [32]. Triggering the TCR of naïve CD45RA^+^ CD4^+^ Tconvs in presence of TGF-β and/or IL-2 results in cells that are highly suppressive and by expressing FOXP3, CD25, CTLA-4 and GITR resemble the phenotype of nTregs [34].

The quality of T cell stimulation is another important determinant for skewing immune responses towards reactivity *versus* tolerance. Suboptimal, non-adequate antigen-presentation and/or co-stimulation by defective (tumor-associated) antigen presenting cells (APCs) as it applies for plasmacytoid DCs [36] or so-called myeloid derived suppressor cells found in patients with ovarian and hepatocellular cancer, promotes iTregs [37].

In analogy to their CD4^+^ counterparts, there exist thymic CD25^+^FOXP3^+^CTLA-4^+^ as well as adaptive CD8^+^ Tregs. They similarly exert immune control by cell-to-cell contact, IL-10, TGF-β and CTLA-4 [36,38]. In a large series of patients with various types of malignancies suppressive IL-10-producing CD8+CD28 cells could be isolated [39] and cells from prostate cancer patients expressing CD28, CD25 and FOXP3^+^ suppressed T cells in a contact-dependent fashion [40].

The DN cells compose 0.8%–1% of the total peripheral T cells and have been shown to suppress in an antigen-specific and dose-dependent manner [41]. Uniquely DN cells exhibit a targeted cytotoxicity against syngeneic CD8^+^ T cells of the same TCR-specificity. Furthermore and in addition to cells with an α/β TCR, CD25^-^FOXP3^-^ γ/δ Tregs that inhibit T cells as well as DCs have been reported [42].

### 2.2. Immunological features

In the past years the modes of Treg-mediated suppression have been diligently studied. A broad repertoire of mechanisms has been discovered, which affect lymphocytes, monocytes and DCs and encompass cell-to-cell contact as well as soluble factors. The importance of cell-to-cell contact is highlighted by the fact that the suppressive activity is abolished *in vitro* once semi-permeable membranes separating Tregs from effector cells are introduced. This observation is furthermore supported by the critical role of the LFA-1/ICAM-1 interaction [43]. On the other hand, IL-10 and TGF-β have been shown to be of substantial importance for the creation of a tolerogenic cytokine environment [44-46], but also for the full suppressive potency of Tregs. In animal studies Tregs derived from IL10^−/−^ and TGF-β^−/−^ mice displayed a substantial lack of suppressiveness. Interestingly Tregs utilize TGF-β not only in its released form to blunt T and NK cell responses, but also bound to their membrane as shown in a study on patients with gastro-intestinal stromal tumors [47]. Of note iTregs isolated from patients with colorectal and head and neck cancer, both inflammation driven malignancies, suppress T cells also in a PGE_2_ dependent manner [48,49].

Recent reports state that IL-35 plays a role for the development as well as (maximal) suppressive activity of Tregs [50,51]. IL-35 is a novel member of the IL-12 heterodimeric cytokine family. A constitutive IL-35 expression could not be found in human nTregs and these findings still require further refinements [52].

It is well established that NK as well as T cells lyse infected or transformed (pre-/malignant) cells by the perforin/granzyme pathway. Astonishingly Tregs are capable of killing effector cells in a uniform fashion [53]. In addition, involvement of Fas ligand, TRAIL-DR5 (tumor necrosis factor-related apoptosis inducing ligand/death receptor) and galectins is suggested as part of this “control-by-killing” strategy [54,55].

Apart from their direct suppressive effects on cells, Tregs are able to modulate the physiological activation of T cells. Very sophisticated *in vivo* models showed that Tregs attenuate the stability of immunological synapses formed between T cells and APCs in the lymph nodes [56]. The affinity of CTLA-4 on Tregs for co-stimulatory CD80/CD86 molecules on APCs exceeds that of CD28 on Tconvs. Binding of CTLA-4 on APCs subsequently leads to a CD80/CD86 downregulation and thereby promotes tolerance instead of reactivity [57]. In addition CTLA-4 mediates an upregulation of indoleamine 2,3-dioxygenase (IDO) in APCs. This molecule metabolizes tryptophan and generates a substantial amount of reactive oxygen species (ROS). Depletion of tryptophan impedes activation and proliferation of T cells while the simultaneously produced ROS exert cytotoxic effects [58]. Recently identified surface molecules important for the Treg-mediated suppression include the lymphocyte activation gene-3 (LAG-3; CD223). It impairs the maturation and immune stimulatory capacity of immature DCs by binding their MHC class II [59]. Neuropilin-1 (Nrp1) on the other hand prolongs the contact (modulation) time of Tregs to DCs. It thereby also restricts the access of effector T cells to the immunological synapse [60].

The suppressive repertoire of Tregs includes a collection of very distinct mechanisms that are summarized in the term metabolic disruption. Extra-cellular adenosine triphosphate (ATP) acts physiologically as a major danger signal. Upon tissue destruction intracellular ATP-reservoirs are released and mediate inflammatory responses through purinergic receptors on immune cells [61]. Tregs express the ectoenzymes CD39 and CD73, which cleave ATP. Thereby they (A) remove the pro-inflammatory stimulus and (B) generate immunosuppressive adenosine. Adenosine suppresses, *via* A2A-receptors activated lymphocytes, promotes iTregs [49,62] and hampers immunogenicity as well as maturation of DCs [63]. In contrast to regular lymphocytes Tregs have high intracellular levels of the inhibitory second messenger cyclic adenosine monophosphate (cAMP), which they can inject into activated effector T cells leading them to anergy [64]. The role of the high CD25 expression on Tregs has been fiercely discussed. CD25 being a component of the IL-2 high affinity receptor (together with CD122 and CD132) leads to the speculation that Tregs may consummate/deplete the local IL-2. This could lead to IL-2 starvation and finally to Bim-mediated apoptosis of (activated) Tconvs [65]. Nevertheless these hypotheses are challenged by the unaltered suppressive function of Tregs upon antibody-mediated blocking of CD25 [66]. Very recent findings describe that Tregs also interfere at the level of the very sensitive redox-metabolism of immune cells. The consumption of thiols and inhibition of the glutathione synthesis in DCs and T cells result in functional alterations as well as in an increased susceptibility towards ROS mediated cytotoxicity [67,68]. These findings are of special importance taking into consideration that cancer and inflammation are associated with oxidative stress and Tregs on their part depict an increased resilience towards ROS [69,70].

### 2.3. Mechanisms Leading to Accumulation in Cancer

**Figure 1.**
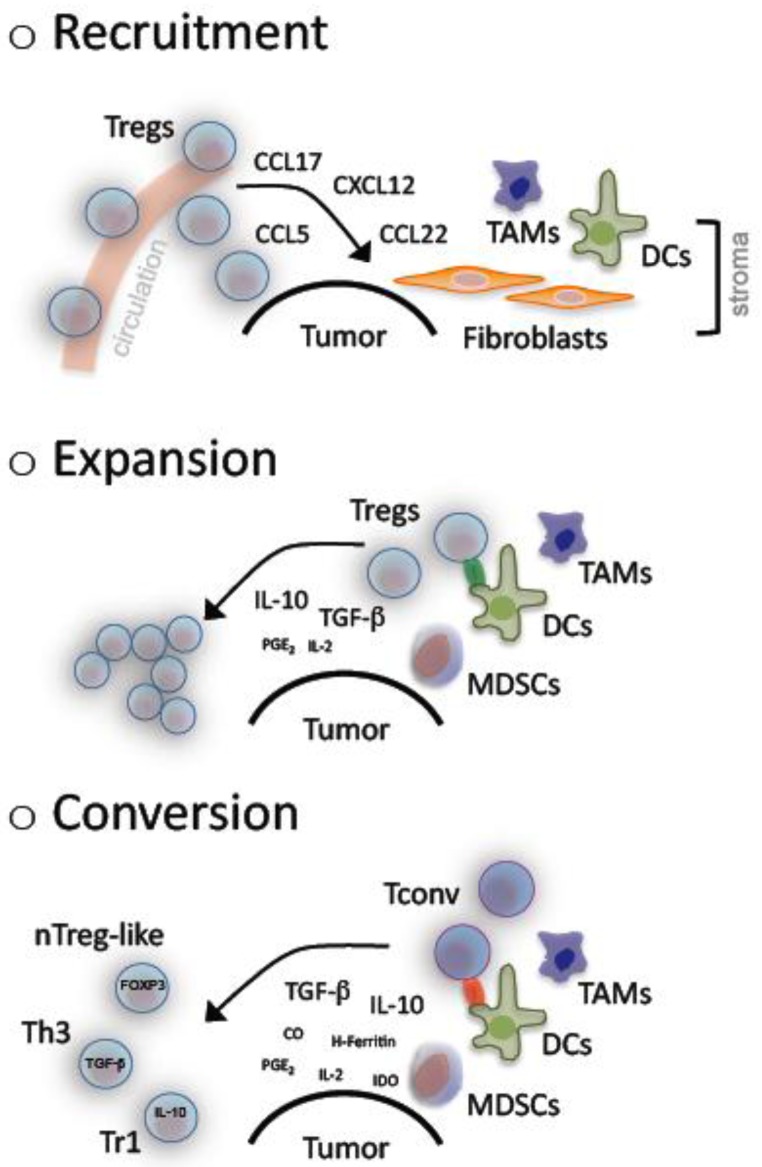
Accumulation of regulatory T cells in cancer. A number of mechanisms lead to the observed accumulation of regulatory T cells (Tregs) in cancer. Malignant cells and/or bystanding fibroblasts, dendritic cells (DCs) as well as tumor associated macrophages (TAMs) in the tumor stroma produce and secrete several chemokines, which are chemoattractive for Tregs and result in their recruitment from the circulation away to the tumor site. Pre-existing Tregs can clonally expand upon antigen-specific activation in presence of mainly TGF-β and IL-10 that are regularly found at high levels within the tumor microenvironment. These two cytokines together with a suboptimal antigen presentation that is provided by tolerogenic DCs, TAMs and/or myeloid derived suppressor cells (MDSCs) additionally promote the conversion of conventional T cells (Tconv) into suppressive, adaptive Tregs including naturally occuring (n) Treg-like, T helper (h) 3 and T regulatory (r) 1 cells.

Accumulation of Tregs has been described in cancer patients for the tumor microenvironment- as well as systemically [32]. As yet *in vitro* as well as *in vivo* observations have revealed a complex underlying system that comprises recruitment, expansion and *de novo* generation of Tregs (Figure 1).

In cancer patients, CCR4^+^ Tregs show a re-directed trafficking towards malignant tissue following a CCL22 chemokine gradient. This was firstly demonstrated in ovarian cancer patients. Interestingly in these patients CCL22 was not produced by the tumor, but released by the “bystanding”, tumor-associated macrophages (TAMs) [23]. In the follow several chemokine receptors were identified on Tregs, among them CCR2, CCR4, CCR5, CCR7, CCR8 and CXCR4. These receptors enable n/iTregs to migrate towards tumors in response to CCL2, CCL5, CCL17, CCL22 and CXCL12 [32,71-74].

Decreased TCR excision circles and an elevated proportion of Ki67^+^ cells strongly indicate an increased turnover of Tregs in cancer patients [74,75]. Several tumors have been shown to release high amounts of TGF-β and/or to induce bystander cells (e.g., MDSCs and immature DCs). These bystander cells represent additional sources of cytokines (e.g., TGF-β, IL-10, PGE_2_ and others) with a fundamental role in Treg expansion as well as maintenance of suppressive features [33,76-78].

Furthermore, and as previously noted, Tregs can be generated from naïve or memory CD4^+^ and CD8^+^ Tconvs. Conditions that favor such a transformation are regularly found in cancer. TGF-β is the key cytokine in such a process while IL-10 holds the secondary role especially for antigen-stimulated cells [78,79]. Other contributing molecules are heme-oxygenase 1 (HO-1), cyclooxygenase 2 (COX-2), H-Ferritin, IDO [58, 81-83] and of course defective APCs [58,80-82].

Altogether it becomes evident that an accumulation of suppressive Tregs results from complex multistep and multifactorial processes, which need to be diligently explored and holistically analyzed in order to develop efficient modes of clinical interventions.

## 3. Regulatory T Cells in Colorectal Cancer

Colorectal cancer (CRC) is one of the leading causes for cancer-related morbidity and mortality in Western countries. Almost half of the patients that receive a curative treatment die from relapse or metastatic disease [24]. The seminal work of Jerome Galon and his colleagues showing that the type, location and density of tumor-infiltrating immune cells are of strong predictive impact supported the hypothesis that adaptive immune responses influence the behavior of human CRC [83,84].

In addition to genetic predisposition and environmental factors, chronic inflammation as for example seen in patients with inflammatory bowel disease (IBD) is linked to an increased CRC incidence following a so-called “inflammation-dysplasia-carcinoma sequence” [85]. The therapeutic and preventive potential of an anti-inflammatory treatment, especially by COX-2 inhibition was early recognized and is currently under clinical evaluation [86]. The concept of an inflammation driven malignant process is further corroborated by increased levels of major inflammatory mediators in CRC-patients resembling systemic inflammatory responses [87]. Microbial intestinal infections during childhood as regularly registered in countries with lower hygienic standards are associated with a lower CRC incidence, which led to the so-called “hygiene theory” [88]. Evidently microbes promote tolerance in an IL-10 dependent manner by amongst others educating Tregs. These Tregs contribute then to the epithelial homeostasis and intestinal integrity, thus preventing inflammation-related malignancies.

**Figure 2.**
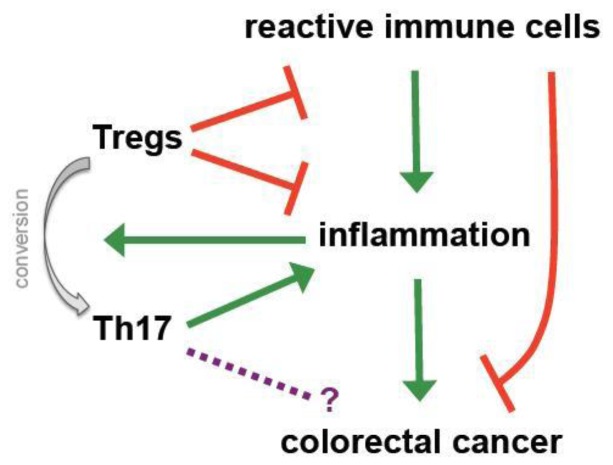
Regulatory T cells in colorectal cancer—friends and foes. The role of regulatory T cells (Tregs) in colorectal cancer is very complex and ambiguous. Chronic inflammation is strongly associated with intestinal carcinogenesis. Tregs efficiently control inflammatory processes and thereby are capable of preventing tumor development by maintaining and restoring intestinal homeostasis. However, under strong inflammatory stimuli they can convert into pro-inflammatory IL-17 producing cells (Th17) cells, which have been linked to cancer initiation. At the same time immunosuppressive Tregs not only contain the inflammatory activity of activated immune cells, but also may hamper their efficient tumor directed response. Taken together these observations suggest a very fine balance between pro- and anti-tumor activities of Tregs in CRC that strongly depend on the examined phase (early *versus* late) of tumorigenesis.

During inflammation physiological countermeasures to limit destruction and to restore homeostasis include the local accumulation of Tregs, which amongst others express CD103 to retain themselves in infected or inflamed regions [26,89,90]. As a consequence more Tregs can be detected in the colon of patients suffering from colitis ulcerosa and IBD than in healthy controls [91] and CD103 expression represents a hallmark of tumor-infiltrating Tregs in (colorectal) cancer [92]. These observations already indicate one of the major controversies regarding the role of Tregs in CRC; they potentially may have a positive impact by controlling cancer-driving inflammation but at the same time they might promote tumor progression by impeding specific immune responses (Figure 2).

### 3.1. Pre-Clinical Animal Models

The paradigm of Tregs being unequivocally detrimental in cancer has been mainly challenged by the pivotal work performed in pre-clinical CRC models [93]. At first an increased susceptibility for inflammatory mucinous cancer was seen in IL-10^−/−^ mice [94]. In APC^Min/+^ mice, in which underlying genetic defects of the β-catenin and wnt-signaling pathway lead to cancer formation [95], adoptive transfer of Tregs and IL-10 administration proved to have protective and even therapeutic effects [96,97]. Furthermore, anti-inflammatory treatment with recombinant IL-10 or neutralizing TNF-α antibodies led to a significant numerical reduction and redistribution of Tregs from the periphery into the centre of regressing malignant lesions [98,99]. As previously mentioned TGF-β is crucial for upregulation and stability of FOXP3. At the same time it positively regulates RORγt, the signature TF for pro-inflammatory Th17 cells that have been related to colitis-induced cancer and the promotion of CRC initiating cells [26,100,101]. Generally FOXP3 inhibits RORγt and the thereby promotes the generation of “classical”, suppressive Tregs [102,103]. However in presence of strong inflammatory stimuli, namely IL-6 and TNF-α, FOXP3 is downmodulated and cell differentiation skewed towards the Th17 pathway. It is speculated that persistent inflammation thereby forms a positive, self-amplifying loop (Figure 2). Suppressive Tregs are transformed into Th17 cells overriding any balance between anti- and pro-inflammatory activities and increasing the risk for a malignant transformation [102,103]. Recent studies suggest that mast cells (MC) are an active partner in this diversion of Tregs during polyposis coli and CRC [104,105]. Interestingly, Blatner and colleagues showed that generation of pro-inflammatory Tregs as well as MC-Treg crosstalk in CRC are both independent of IL-6 and IL-17 [105]. These findings suggest the existence of an alternative, to the classical Th17 conversion, transformation of anti- to pro-inflammatory Tregs. For the future it will be very interesting to investigate the therapeutic potential of such MC-Treg interaction in CRC.

### 3.2. Prognostic Value of Regulatory T Cells

One of the first studies in CRC patients showed that peripheral blood derived CD4^+^CD25^+^ T cell lines inhibited *via* TGF-β cytotoxicity and proliferation of autologous HLA-A1 restricted CD4^+^ CTLs [106]. An early quantitative analysis of circulating CD4^+^CD25^+^ Tregs in patients with epithelial cancers, including nine CRC cases, revealed increased frequencies. These cells expressed CTLA-4, produced TGF-β and impaired both, T and NK cell functions [107]. In the follow several studies confirmed the findings on the elevated proportions of CD4^+^ Tregs in peripheral blood [108-111].

Initial analyses of TILs evidenced increased levels of CD4^+^ Tregs compared to autologous healthy tissue without any clear correlation to disease stage [108,111,112]. Tregs infiltrated predominantly the tumor stroma resulting in a reversal of the stroma/epithelium ratio seen in healthy tissue [112]. The number of Tregs was two-fold higher in limited as compared to metastatic disease. This observation led to the not yet verified speculations that Tregs may migrate from the primary lesion towards the metastases during disease spreading. The positive correlation of stromal DCs with infiltrating Tregs implies a potential link between both populations [113]. In line with the other examined compartments CD4^+^CD25^high^FOXP3^+^CTLA-4^+^ Tregs were also increased in the tumor draining mesenteric lymph nodes (TDLNs) [110]. Noticeably Tregs from TDLNs can express COX-2 by which they suppress IFN-γ as well TNF-α production of peripheral T cells; thus providing an additional argument in favor of COX-2 inhibition in CRC patients [48].

Several groups have investigated the impact of Tregs on the specific immune responses against tumor-associated antigens (TAAs) in CRC. The grade of local infiltration did not correlate with responses against well-defined TAAs as EpCAM, Her-2/neu and CEA [113]. Depleting Tregs in PBMCs from CRC patients dramatically boosted the IFN-γ and TNF-α production in T cells, which were stimulated with a CEA peptide [48]. In spite the unmasking of responses against several other TAAs, recall antigens like PPD and HA were not affected suggesting a TAA-specific rather than a systemic immune suppression [110]. In a very comprehensive analysis various TAA-specific Tregs were exclusively identified in CRC patients. Peptides for CEA, telomerase, Her-2/neu and MUC-1 all led to an activation of Tregs [114]. TAA-specific Tr1 cells were successfully identified using a p53 peptide [115]. In addition to CD4^+^ Tregs also CD8^+^CD28^-^ Tregs could be isolated from peripheral blood, tumor tissue and metastatic lymph nodes of CRC patients [39]. These cells suppressed T cells in an IL-10 dependent fashion and were mainly CCR4^+^, which may have contributed to their accumulation *via* recruitment. A recent study identified circulating and tumor infiltrating CD28^+^ CD8^+^ Tregs with a CD25^+^, FOXP3^+^, CTLA-4^+^, GITR^+^, CCR4^+^, TGF-β^+^ and CD127^low/neg^ phenotype [111]. Remarkably this type of Tregs was found in 90% of the CRC specimens but was totally absent in normal colonic tissue suggesting a cancer-specific presence without contribution to the physiologic epithelial homeostasis [90]. Ligands for CCR4 (e.g., CCL17 and/or CCL22) were in contrast to IL-6 and TGF-β not highly expressed in the tumor tissue, altogether indicating a conversion from CD8^+^ Tconvs rather than a tumor directed migration as the cause for the observed infiltration. In another recent study CXCL11 produced by CRC-derived CD68^+^ myeloid cells is suggested to be a promising chemoattractant for Tregs [116].

Among CRC 10%–15% of the cases are characterized by high levels of micro-satellite instability (MSI-H) that results from defective DNA mismatch repair (MMR)-systems. These types of CRC depict an increased infiltration of activated immune cells and have a relatively better prognosis [117]. The augmented immune response is ascribed to the highly immunogenic frame-shift derived TAAs. A stratification based on MSI status revealed that patients with MMR deficiency had a significantly higher infiltration with FOXP3^+^ CD4^+^ Tregs potentially as result of the pronounced immune response and its accompanying controlling components [118]. These findings were similar to observations by Sinicrope and Nosho [119,120], but in opposition to a study, which assessed FOXP3 mRNA levels instead of staining for cells [121]. Differences in the patients groups and more importantly in the applied methodologies may account for the varying observations and interpretations. It should be taken into consideration that FOXP3 mRNA can be present without translation and a potential FOXP3 expression by tumor cells can severely influence the results.

In a clinical trial for CRC patients combining chemotherapy (gemcitabine plus FOLFOX) with immune stimulants (granulocyte macrophage colony-stimulating factor and interleukin-2) increased levels of circulating Tregs almost normalized after two treatment cycles. The drop in Tregs was accompanied by an increase in T cell responses against tumor cell lysates, a patients' response-rate of almost 70%, and restoration of the CD4^+^/CD8^+^ T cell ratio [109]. This data could represent a strong indication for the clinical relevance of Tregs for anti-tumor responses and the potential benefit of their removal. However, it cannot be precluded that the beneficial effects were confounded phenomena and it still remains unsettled whether Tregs exert a substantial inhibitory *in vivo* effect in CRC patients.

In analogy to the numerous studies on the prognostic impact of Tregs in various types of solid tumors [32], for which the presence of Tregs is mostly associated with a worse prognosis, several groups are interested in their predictive value for CRC (selected studies are summarized in Table 1). At first, research focused on the proportion of Tregs among the TILs. Most studies were carried out by immunohistochemistry (IHC) using the FOXP3 antibody clone 236A/E7 that has been extensively tested and shown to detect mostly CD4^+^CD25^+^ Tregs [11]. However, it has to be emphasized that enumeration and visualization techniques (FOXP3 single- *versus* co-staining with other surface molecules, e.g., CD4) as well as the chosen FOXP3 clone have been shown to substantially influence the results making further standardization obligatory in order to achieve direct inter-study comparability [122,123]. In a French study on a large data set (n = 967) high density of Tregs within the tumor and weak infiltration of the surrounding healthy tissue was an independent positive prognostic marker [124]. The observed beneficial effect of intratumoral Tregs is in contrast to most findings in other solid cancers [32]. It reflects however very well the conclusions drawn from several pre-clinical models [93,96,97,99,125]. Similar observations were made in a cohort of 1420 patients, in which strong infiltration with Tregs was linked to a better survival, especially in MMR-proficient CRC [126]. In a side study of a phase-3 trial for relapsed CRC patients comparing standard FOLFOX-4 treatment with GOLFIG-2, high up-front infiltration with Tregs was of positive prognostic value for treatment response as well as for survival [127].

As described previously Tregs infiltrate more preferably the tumor stroma [108,112]. A higher stromal infiltration has been associated with limited disease and less metastatic lymph nodes [112,119]. Despite the rather low infiltration, the epithelial CD3/FOXP3 ratio had a significant impact for the five-year disease free survival (DFS). It even exceeded the prognostic potency of metastatic lymph nodes and TNM [119]. Similarly an increased CD8/Treg ratio (without discriminating between epithelial and stromal infiltrates) was an independent positive predictor for a better overall survival (OS) in patients undergoing curative surgery [128].

In TDLNs tumor antigens are firstly presented to cells of the adaptive immune system initiating a specific anti-tumor response. Since they simultaneously also represent the preferential site for CRC-metastases, TDLNs are an immunological compartment of great interest. In a study evaluating TDLNs from patients that underwent a radical resection, CD4^+^CD25^+^FOXP3^+^ Tregs correlated with disease stage and functional alterations of the adjacent CD8^+^ T cells. Suppression was reverted *in vitro* by depleting the Tregs [129]. However high Treg infiltration in sentinel lymph nodes appears to be associated with a better survival as indicated in a study that includes a rather limited number of cases (n = 30) [130] and further larger scale investigations in TDLNs remain mandatory.

## 4. Targeting Regulatory T Cells in Cancer

Based on a multitude of reports from mainly murine tumor models including also CRC [131], *in vivo* Treg depletion may be capable of enhancing anti-tumor responses in patients thus being clinically effective [132]. As discussed previously in detail, studies regarding the role of Tregs in CRC on disease course and outcome have been controversial. The majority of them currently points towards a beneficial rather than deleterious function. Although this dispute is still ongoing and attempts to target Tregs in CRC patients should therefore be performed very cautiously, some of the actual candidates to overcome Treg-mediated suppression will be shortly discussed.

In this context, among all the chemotherapeutics cyclophosphamide, which in low or metronomic dosages selectively obliterates Tregs while preserving effector T cells has been the most actively investigated agent. As yet only modest effects have been presented clinically and the transient reduction also comprises the risk of a rebound in Treg frequencies exceeding even the pre-treatment levels [133,134]. Other promising strategies include targeting of Tregs directly through the surface molecules that they predominantly express. One of the initial target molecules was CD25, which however is not expressed on all FOXP3^+^ Tregs and more importantly is also found on activated effector T cells. A recombinant fusion protein composed of IL-2 and diphtheria toxin (denileukin diftitox) that is directed against CD25 expressing cells was the first to show an efficient reduction of Tregs in (colorectal-) cancer patients [135,136]. In a phase 1 study including 15 CRC patients, the combination of denileukin diftitox treatment with DC vaccination led to a promising enhancement of the specific immune response against CEA [136]. Several other anti-CD25 antibodies with or without coupled immunotoxins (e.g., basiliximab and daclizumab) are currently under clinical evaluation. The short-lasting activities of these agents as well as the robust Treg-homeostasis still represent considerable hindrances [137,138].

The negative immune modulator CTLA-4 is physiologically found on activated immune cells delivering inhibitory signals during activation [139]. Especially nTregs constitutively express CTLA-4 and its blockade by non-depleting humanized monoclonal antibodies ipilimumab and tremelimumab has the potential to restore anti-tumor immunity. Promising immunological as well as clinical responses especially in melanoma have been reported [140-142]. Notably anti-CTLA-4 treatment is associated with severe autoimmune effects, such as dermatitis, colitis and hepatitis, emphasizing the potential risk of inducing autoreactivity by manipulating Tregs.

As Tregs express various toll-like-receptors (TLRs) the according ligands may positively or negatively alter their function [143]. This fact is of great interest for immunotherapies, since several TLR stimulators, among them CpG oligodeoxynucleotides and imiquimod, are already used as adjuvants to boost vaccine responses within clinical trials.

Alternative approaches to inactivate Tregs directly or indirectly include *inter alia* targeting of GITR, B7-H1, COX-2, TGF-β and the chemokine mediated migration.

## 5. Concluding Remarks

Research over the past 20 years has proven that Tregs hold a prominent role in tumor immunology and, in particular, within the process of immune escape. There is convincing evidence that Tregs inhibit anti-tumor activity and *in vitro* depletion of Tregs successfully unmasked several TAA-directed responses in CRC patients. Nevertheless it still remains to be conclusively clarified whether these impeding effects exerted by Tregs on immune responses are not (over-) compensated by their beneficial actions on inflammation. A large body of observations from pre-clinical as well as clinical studies suggests that inflammation promotes tumor growth and progression in CRC. Consequently anti-inflammatory Tregs possess the potential to hinder CRC progression as Erdman and colleagues have shown in several very elegant murine studies. However, a number of additional eventualities must be taken into consideration when patients are involved; regular, suppressive Tregs can, under certain environmental conditions, be transformed into pro-inflammatory Th17 cells, which could of course promote inflammation-driven cancers or, contrarily as has been shown for other types of cancers, even target the malignant cells. Furthermore, it may be speculated that initially Tregs may hinder tumor growth by controlling inflammation as seen in several murine models, but after the establishment of a certain tumor mass, not critically dependent on pro-inflammatory stimuli, they may turn against the host by hampering an efficient adaptive anti-tumor response. At this latter stage, they could represent a therapeutic target for favorably boosting immunity (Figure 2). These intriguing and complex issues remain to be unequivocally answered before contemplating if, when and how to target Tregs in CRC patients.

The attempts to characterize Tregs in CRC patients are rather “young”. Standardization in methodologies, the analyzed compartments and patients groups is required to ensure proper comparability between the various studies. Beyond the documented numerical alterations functional properties of the patient's Tregs need to be addressed more in detail. In addition to the cells with a naturally occurring (-like) phenotype the induced Tr1 and Th3 forms have to be analyzed more specifically since the CRC microenvironment contains high levels of several of the “ingredients” (e.g., IL-6, PGE2, TGF-β) needed for a successful conversion of Tconvs into iTregs. Up to date and despite the comprehensive analyses regarding the impact of Tregs, TNM classification remains the best guide to base the decisions on the patient's management on. Assessment of Tregs in its present form is far from being routinely incorporated into diagnostic as well as decision making procedures. For the future it will be critical to evaluate prospectively in the context of clinical trials whether and how presence of Tregs in the various compartments can predict the response to a particular therapy.

Taken together Tregs possess a very complex role in CRC and revealing their network as well as their spatial and temporal impact may help to better understand the pathophysiology in order to modulate them in a beneficial way for the patients.

## Figures and Tables

**Table 1.** Clinical Studies on the Impact of Regulatory T Cells in Disease Course and Outcome of Colorectal Cancer.

**Author/Journal/Year**	**Patients Included**	**Method**	**Compartment**	**Major Conclusions**
Loddenkemper *et al.* Journal of Translational Medicine 2006 [110]	40	IHC	Primary CRC	(1) No difference in survival in patients with high and low total FOXP3 infiltration (2) Weakly better survival in patients with higher epithelial Treg infiltration
Salama *et al.* Journal of Clinical Oncology 2009 [120]	967	IHC	Primary CRC	(1) Intratumoral FOXP3 density associated with better survival (2) High FOXP3 infiltration of normal mucosa associated with worse survival
Sinicrope *et al.* Gastroenterology 2009 [117]	160	IHC	Primary CRC	(1) Low intraepithelial CD3/FOXP3 is a independent marker for reduced 5-year DFS (2) CD3/FOXP3 stronger prognostic variable than tumor stage and LN number
Suzuki *et al.* Cancer Immunology and Immunotherapy 2010 [124]	95	IHC	Primary CRC	(1) High intratumoral CD8/FOXP3 independent prognostic marker for OS in patients undergoing curative surgery (2) Tumor TGF-β is implicated in FOXP3+ cell accumulation
Frey *et al.* International Journal of Cancer 2010 [122]	1420	IHC	Primary CRC	(1) High FOXP3 infiltration independent positive predictor for 5-year DSS in MMR-proficient patients (2) FOXP3 density not of prognostic value for MMR-deficient patients
Correale *et al.* Journal of Immunotherapy 2010 [123]	57	IHC	Primary CRC	(1) High FOXP3 infiltration associated with better OS (2) High FOXP3 infiltration predicted better treatment response (GOLFIG-2/FOLFOX-4)
Deng *et al.* Clinical Cancer Research 2010 [125]	34	FACS	Primary CRC/PB/MLN	(1) Proportion of Tregs in TDLN were > PB and < TIL (2) Infiltration of FOXP3+ cells correlated with disease stage
Matera *et al.* Gut 2010 [126]	30	IHC	MLN	(1) High FOXP3 levels in SLN associated with early disease stage (2) Better survival in patients with higher FOXP3 frequencies
Nosho *et al.* Journal of Pathology 2010 [118]	768	IHC	Primary CRC	(1) FOXP3 infiltration correlated with survival (2) FOXP3 is not a independent prognostic marker

Abbrevations: IHC: immunohistochemistry; CRC: colorectal cancer; Treg: regulatory T cells; DFS: disease free survival; LN: lymph nodes; OS: overall survival; MMR: mismatch repair; FACS: flow cytometry; PB: peripheral blood; MLN: mesenteric lymph node; TDLN: tumor draining lymph node; SLN: sentinel lymph node.

## Abbreviations

APC: antigen presenting cell
ATP: adenosine triphosphate
cAMP: cyclic monophosphate
CEA: carcinoembryonic antigen
CRC: colorectal cancer
CTL: cytotoxic lymphocyte
CTLA-4: cytotoxic T lymphocyte antigen 4
DFS: disease free survival
DN: double negative
EpCAM: epithelial cell adhesion molecule
FOLFOX: folinic acid, fluorouracil, oxaliplatin
FOXP3: forkhead box P3
GITR: glucocorticoid induced TNFR-related protein
GOLFIG: gemcitabine, oxaliplatin, levolonic acid, fluorouracil, GM-CSF, IL-2
HA: hemagglutinin
Her2/neu: human epidermal growth factor receptor 2
HLA: human leukocyte antigen
HPF: high power field
IBD: inflammatory bowel disease
ICAM-1: intercellular adhesion molecule
IDO: indoleamine 2,3-dioxygenase
IHC: immunohistochemistry
iTreg: induced regulatory T cell
LFA-1: lymphocyte function associated antigen 1
MC: mast cell
MDSC: myeloid derived suppressor cell
MHC: major histocompatibility complex
MMR: mismatch repair
mRNA: messenger ribonucleic acid
MUC-1: mucin 1
MSI-H: microsatellite instability high
nTreg: naturally occurring regulatory T cell
OS: overall survival
PBMC: peripheral blood mononuclear cell
PPD: purified protein derivate
ROS: reactive oxygen species
TAA: tumor associated antigen
Tconv: conventional T cell
TCR: T cell receptor
TDLN: tumor draining lymph node
TF: transcription factor
TIL: tumor infiltrating lymphocyte
TLR: toll like receptor
TNM: tumor/node/metastasis
Treg: regulatory T cell
